# The Role of Stress and Cognitive Absorption in Predicting Social Network Addiction

**DOI:** 10.3390/brainsci12050643

**Published:** 2022-05-13

**Authors:** Loreta Cannito, Eugenia Annunzi, Caterina Viganò, Bernardo Dell’Osso, Matteo Vismara, Pier Luigi Sacco, Riccardo Palumbo, Claudio D’Addario

**Affiliations:** 1 Department of Psychological, Health and Territorial Sciences, University “G. d’ Annunzio” of Chieti-Pescara, 66100 Chieti, Italy; loreta.cannito@unich.it; 2 Center for Advanced Studies and Technology, University “G. d’ Annunzio” of Chieti-Pescara, 66100 Chieti, Italy; 3 Department of Neuroscience, Imaging and Clinical Sciences, University “G. d’ Annunzio” of Chieti-Pescara, 66100 Chieti, Italy; eugenia.annunzi@gmail.com; 4 Department of Biomedical and Clinical Sciences Luigi Sacco, University of Milan, 20100 Milan, Italy; caterina.vigano@unimi.it (C.V.); bernardo.dellosso@unimi.it (B.D.); matteo.vismara@unimi.it (M.V.); 5 Department of Psychiatry, ASST Fatebenefratelli Sacco, University of Milan, 20100 Milan, Italy; 6 Department of Philosophical, Pedagogical and Economic-Quantitative Sciences, University “G. d’ Annunzio” of Chieti-Pescara, 66100 Chieti, Italy; pierluigi.sacco@unich.it; 7 MetaLAB (at) Harvard, Cambridge, MA 01451, USA; 8 ISPC-CNR, 80134 Naples, Italy; 9 Faculty of Bioscience and Technology for Food, Agriculture and Environment, University of Teramo, 64100 Teramo, Italy; 10 Department of Clinical Neuroscience, Karolinska Institutet, 17177 Stockholm, Sweden

**Keywords:** social network addiction, stress, cognitive absorption, temporal dissociation, internet addiction

## Abstract

Nowadays, the use of social networks (SNs) is pervasive and ubiquitous. Among other things, SNs have become a key resource for establishing and maintaining personal relationships, as further demonstrated by the emergence of the pandemic. However, easy access to SNs may be a source of addictive behaviour, especially among the younger population. The literature highlights various psychological and physiological factors as possible predictors of vulnerability to SN addiction. This paper explores the joint effects of stress level and cognitive absorption, in the form of temporal dissociation while on SNs, on the addiction of university students to SNs. Here, 312 participants were involved in an online survey. About 14% of the sample presented a risk for SN addiction. Moreover, it was found that stress level predicted SN addiction both directly and indirectly through the effect of individual temporal dissociation, as experienced during SN usage. These results suggest a significant role of perceived stress level on addiction risk, while also pointing out additional vulnerability to SN addiction for cognitive profiles that are relatively more prone to temporal dissociation while online.

## 1. Introduction

The introduction of social media, such as Facebook, Instagram, and Twitter, has brought about dramatic changes in interpersonal communication and relationships [1]. With the exponential increase in the number of active social media users [2], there has been a parallel reduction in engagement in other forms of communication, such as phone calls, email correspondence, and face-to-face interaction [3], as well as in the use of older legacy media such as books, TV, and movies [4]. Social networks are web-based services that enable the pursuit of several different goals, including, among others, building one’s public or semi-public persona in the digital space, defining a personal network of relations with varying degrees of (e-)proximity, and gaining insight into and access to the personal networks of others [5]. In view of the well-known hyper-sociality of human beings [6], social networks and (online) social presence have unsurprisingly become a central sphere of activity in the life of many individuals. SNs may sometimes be associated with beneficial effects on human relations, as in the case of people with poor face-to-face sociability [7]. The potential benefits of SNs became even clearer during the COVID-19 pandemic, in which social distancing caused digital relationships to become, at times, the only viable option [8]. Even now that restrictions are gradually being lifted despite the pandemic not being over yet, the digital sphere of relations still maintains a key relevance, as people’s interaction modes and habits have permanently adjusted to the new status quo [9]. On the other hand, the pervasive presence of, and ease of access to, social media and the strong social incentives to take part in online conversations, which provide increasingly well understood biological, cognitive, and social rewards [10], pave the way to possibly dysfunctional and even addictive practices of social media use in the general population, particularly among younger individuals [11]. As the business model of social media calls for encouraging users to stay online as long as possible by fuelling a constant sense of anticipation of forthcoming rewards [12], addictive behaviours are generally not purposefully prevented by the interaction design of digital platforms, and are possibly even encouraged by features such as the dynamic scrolling of content [13] and push notifications [14], not to mention the possibility of favouring further substance addictions in turn [15]. However, a comprehensive theoretical understanding of the processes through which SN usage leads to addiction is still lacking [16].

It is still an open point whether such new forms of addiction should be included in DSM-5 [17], which currently refers to them as a “condition for further study”. However, there is evidence suggesting that the psychological processes of people affected by internet/social network addictions are characterized by, among others, increased salience of the addictive activity, mood modification, craving, withdrawal, and functional impairment—all of which are common criteria that jointly define substance abuse disorders [18]. Subjects with internet/social network-related addictive behaviours develop specific forms of anxiety, feel stressed by deprivation, and fail to successfully control their access time, but also integrate online activities into their coping strategies to manage negative affect, to secure buffering rewards, and to exploit them as an excuse for the procrastination of other less pleasant activities [19,20,21]. Almost inevitably, a major consequence of the COVID-19 pandemic has been that of further exacerbating such practices in addicted subjects, and of spreading them further across the population [22,23], with an overall increase in the incidence of related addictive behaviours [24]. The social relevance of the phenomenon and its potentially dysfunctional impact on human conduct [25] have led to it being flagged as a public health issue of serious concern [26]. Significantly, in a large-scale dataset, a weak association was found between all-purpose digital technology use and adolescent well-being [27]. However, a series of variables, such as gender, age, personality traits, and specific clinical conditions, among others, are also likely to play a significant role in the prediction of excessive use and susceptibility to addiction [28]. 

In particular, the role of stress appears to be of special importance. For example, Feng et al. reported that social anxiety partially mediates the impact of stress on internet addiction in adolescents [29]. Similarly, Jun and Choi, analysing data on stress in educational environments, concluded that adolescents who endure scholastic stress, especially when accompanied by negative affect, may be at higher risk of internet addiction [30]. Analogous results have been reported by Brailovskaia et al., who highlighted that daily stress is positively linked to propensities towards specific SN addictions (especially Facebook) [31].

Based on this evidence, and in view of the concomitant increase in the stress levels recorded in the population since the beginning of the pandemic [32], the first aim of the current work is to assess the direct influence of perceived stress during the pandemic on participants’ addiction to SNs. We therefore hypothesized that the perceived stress level positively predicts the addiction level. 

Nonetheless, recent literature on the impact of the COVID-19 pandemic on cognitive processing has shown that prolonged experience of lockdown and, more generally, major changes in everyday habits cause a distortion of subjective time perception [33,34]. For example, it has been reported that a slower subjective perception of the passage of time is associated with increased levels of stress among the U.K. population [33]. In addition, in a longitudinal study on the French population, Droiet-Volet et al. reported that the improvement in the dynamic perception of subjective experience (in terms of faster-paced time flow, countering the annoyance of excessively slow perceived passage of time) during the confinement period was related to a decrease in the level of boredom as the lockdown progressed [34]. Furthermore, as time spent on SNs is one of the key markers of the severity of addiction [35], we considered whether the perceived stress level impacts temporal dissociation while on SNs (by exacerbating the annoying perception of a too slow passage of time), thus playing a role in the relationship between stress and SN addiction. We therefore hypothesized that temporal dissociation during SN usage, as an indicator of longer time spent on SNs, would positively predict the addiction level.

When considering existing evidence on the relationship between stress and dissociative processes, if the individual perceives a high level of stress, dissociation may work as a coping strategy to mitigate this perception. This idea is supported by findings investigating different kinds of stressful experiences (e.g., early life stress [36] and daily stress [37]). On the other hand, in agreement with earlier literature on addiction (e.g., [38,39]), an increasing amount of evidence suggests that, the higher the stress level, the higher the susceptibility to addiction. Moreover, as time spent is a key marker to predict internet/social network addiction (e.g., [40]), and as higher temporal dissociation may be conducive to underestimating the amount of time spent online, we expect that this may result in the temporal dissociation level positively predicting addiction level.

To flesh out in some more detail the construct of temporal dissociation and the related time distortion, we refer to the construct of cognitive absorption, defined as a state of deep involvement with online experience [41]. A number of studies have found that cognitive absorption is positively associated with internet addiction/problematic use [42,43]. This applies even more for social network addiction [44]. According to the cognitive absorption model proposed by Agarwal and Karahanna, cognitive absorption is described by five dimensions: focused immersion, engagement, control, curiosity, and temporal dissociation [41]. The latter factor is defined as the “inability to register the passage of time while being engaged in the interaction with a device” [41]. Although, from a functional point of view, cognitive absorption has been mainly studied as related to different domains, such as, technology acceptance or e-learning environments [45,46], little evidence is available about the role of cognitive absorption in digital addiction. Regarding temporal dissociation and problematic technology use, a significant time distortion during the use of smartphones has been found by comparing actual and estimated usage time as declared by participants, with a larger distortion reported by participants who spent more time on their smartphone [47]. To the best of our knowledge, there is only one other paper that studies the impact of cognitive absorption on both smartphone and SN addiction [44]. However, no results are available so far about the role of cognitive absorption, and specifically of temporal dissociation, as a third intervening variable influencing the relationship between perceived stress and SN addiction. Therefore, the second aim of the current work is to evaluate whether different levels of temporal dissociation while on SNs bring about a different impact of perceived stress on SN addiction.

## 2. Materials and Methods

### 2.1. Participants

A sample of 312 voluntary participants (male = 30.5%; mean age: 20.9 ± 2.7 SD) was recruited through public announcement at college classes. Participants were recruited from undergraduate programs across different majors. The recruitment call specified that only active social media users could take part in the study. 

### 2.2. Procedure

The research complies with the Declaration of Helsinki and received approval from the appropriate Ethics Committee (Local Ethics Committee Regione Abruzzo ASL 1 Protocol #0008934/20, 14/01/2020 int 271). Participants, after providing their informed consent, completed an online survey and received no monetary or credit compensation for their participation in the study. A power analysis on an R package, WebPower [48], was performed, and the results suggested a good power for each path (≥0.8). The survey included demographic questions (age and gender) and three psychometric scales in order to measure (i) perceived stress level, (ii) temporal dissociation as induced by cognitive absorption while on SNs, and (iii) SN addiction. Data were collected through a web-based platform (Qualtrics, Provo, UT, USA). All of the collected data were analysed through SPSS 22.0, by performing Pearson’s correlation and independent *t*-test analyses. All of the required assumptions were met. The mediation model was tested using Model 6 in PROCESS, an SPSS macro for mediation, moderation, and conditional process modelling [49].

### 2.3. Perceived Stress Scale (PSS-10)

The Perceived Stress Scale (PSS) is an established diagnostic tool that is commonly used to measure stress levels, especially in research focused on the role of stress in the aetiology of diseases and behavioural disorders [50]. For the purpose of the current work, the Italian 10-item version of the scale was administered (PSS10) [51]. Respondents were asked to answer to questions about their self-assessed psychological state during the last month using a five-point Likert scale (from never = 0 to very often = 4). An example of an item is, “How often have you been upset because of something that happened unexpectedly?”. The measure had a good internal consistency in the present sample (α = 0.79).

### 2.4. Bergen Social Media Addiction Scale (BSMAS)

To measure SN addiction, the six-item Bergen Social Networking Addiction Scale (BSNAS), an adaptation of the Bergen Facebook Addiction Scale (BFAS), where the word “Facebook” is replaced with “social media”, was administered [52,53]. The scale incorporates the theoretical framework of the addiction components of the biopsychosocial model [54]. The BSMAS was developed by selecting the six items with the highest possible factor loadings for each component (i.e., salience, mood modification, tolerance, withdrawal symptoms, conflict, and relapse) from a pool of 18 initial items. The six selected items were answered on a five-point scale ranging from very rarely (1) to very often (5), thus yielding a sum score from 6 to 30 [55]. An example of an item is, “I spend a lot of time thinking about social media or planning how to use it”. This scale was validated with different samples, including non-student adults [56]. Such scales measure a person’s level of addiction, and a cut-off score marks the threshold beyond which subjects can be considered at risk of social media addiction [57,58]. Even though there is no specific cut-off score for this scale, the research suggests setting it at 19 for optimal separation of at-risk subjects from low- or no-risk ones, as highlighted by Bányai et al. (2017), by means of sensitivity and specificity analysis [59]. In the present sample, the Cronbach’s alpha of the BSMAS was = 0.85. 

### 2.5. Cognitive Absorption Scale

To measure the level of cognitive absorption while on SNs, the Cognitive Absorption Scale 25 was adapted by suitably inserting “using social media apps” in each item, where appropriate. All of the construct items were measured on seven-point Likert scales from 1 = strongly disagree to 7 = strongly agree. The scale allows for assessing five factors: focused immersion (the experience of total engagement where other attentional demands are, in essence, ignored), heightened enjoyment (the pleasurable aspects of the interaction), control (the user’s perception of being in charge of the interaction), curiosity (the extent to which the experience arouses an individual’s sensory and cognitive curiosity), and temporal dissociation (the inability to register the passage of time while engaged in interaction), which was the factor of interest for the current study. An example of an item measuring temporal dissociation is, “Time appears to go by very quickly when I am using social networking apps on my smartphone”. For the present sample, α = 0.82 was found.

## 3. Results

### 3.1. Descriptive Statistics and Gender Differences

About 14% of the sample reported a score indicating a risk of SN addiction (BSMAS score ≥ 19). Pearson correlation coefficients were computed to assess the relationship between stress level, cognitive absorption (total score), temporal dissociation from cognitive absorption scale, and SN addiction (Table 1). SN addiction was positively correlated with temporal dissociation (r = 0.503, *p* < 0.001), as well as with stress level (r = 0.292, *p* < 0.001) and cognitive absorption total score (r = 0.620, *p* < 0.001). A positive correlation was also found between temporal dissociation and stress level (r = 0.320, *p* < 0.001) and between stress level and cognitive absorption total score (r = 0.248, *p* < 0.001).

Then, in order to investigate gender differences in the study variables, a series of *t*-tests on the whole sample were performed with Bonferroni correction for multiple comparison (with adjusted α = 0.016). The inspection of Q−Q plots revealed that all of the dependent variables were normally distributed for both groups. For all of the three tested variables, Levene’s test was found to be not significant, confirming the homogeneity of variance. The results revealed a significant difference for two of the three variables, with female participants reporting a significantly higher perceived stress level and significantly higher temporal dissociation compared with male participants (see Table 2). A not significant *p*-value (*p* = 0.07) was detected when comparing male and female participants on SN addiction scores. 

### 3.2. Mediation Model

**Figure 1:**
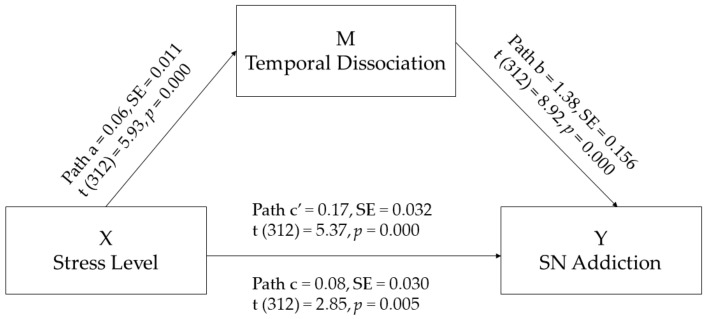
Mediation model. SE, standard error.

A mediation model was then run to study the direct and indirect effects of X, the independent variable (perceived stress level), on Y, the dependent variable (SN addiction), while also modelling a process wherein X causes Y through the intervention of a third variable M (temporal dissociation). In this model, perceived stress level is assumed to influence temporal dissociation during SN usage, which then influences SN addiction. As illustrated in Figure 1, the total effect (path c) is the sum of the direct (path c’) and indirect (path ab) effects of stress level on SN addiction. The model was run allowing bootstrapping with 5000 samples. Bias-corrected point estimates for the indirect effects of the stress level on SN addiction were calculated, together with standard errors and 95% confidence intervals (see Figure 1). A significant direct link between stress level and SN addiction (path c’) was detected, with a higher stress level predicting more serious SN addiction. Furthermore, the results revealed that stress level directly predicts temporal dissociation while on SNs (path a), suggesting that, the higher the participants’ stress level, the more they tend to feature temporal dissociation while on SNs. Moreover, temporal dissociation during SN usage was found to significantly predict SN addiction (path b), which indicates a significant role of cognitive absorption in influencing university students’ risk of SN addiction (see Table 3). Finally, the total effect model was significant; F (2, 309) = 57.9, *p* = 0.000, R^2^ = 0.27. 

## 4. Discussion

The purpose of this paper was, primarily, to evaluate the influence of perceived stress on SN addiction in a sample of university students during the COVID-19 pandemic. SN/internet addiction is currently a major public health issue. It has been shown, for instance, that limiting the internet use of individuals with problematic online access habits has physiological and behavioural effects akin to those of abstinence from sedative or opiate drugs [60]. The issue seems to have been exacerbated by COVID-19-related lockdown restrictions, which turned online interaction into the only viable option for many [61,62]. In view of its major relevance, the topic of social media behaviour and its related pathologies has been investigated from several disciplinary angles [63], with a view to developing effective strategies to cope with problematic and socially critical situations [64]. Our paper, in line with the existing literature [43], did not find significant gender differences in SN addiction. However, significant differences in perceived stress level existed between male and female participants. This result is aligned with evidence of a higher perceived stress level in female college students during lockdown [65]. We also tested whether temporal dissociation due to cognitive absorption while on SNs mediated the relationship between perceived stress and SN addiction. As shown by the mediation model results, perceived stress was found to be a significant positive predictor of SN addiction, as well as of temporal dissociation. Moreover, temporal dissociation as a result of cognitive absorption was shown to predict SN addiction as well, in line with the results presented in another study where the relationship between cognitive absorption and SN addiction was analysed through structural equation modelling [66]. Thus, the results of the mediation model suggest that higher levels of perceived stress directly predict a stronger addiction to SNs, but also that, when feeling more stressed, there is an increased risk of temporal dissociation while on SNs—which, in turn, affects SN addiction. Our findings seem to support a possible functional interpretation of temporal dissociation as a cognitive coping strategy deployed to regulate internal and external stressors, as also suggested by the result that perceived coping self-efficacy directly and negatively predicts dissociation [67]. 

Our results agree with previous research showing that temporal dissociation predicts problematic internet use [68] and that temporal dissociation is conducive to a longer time spent online, which is, in turn, associated with depression [11]. It is then possible to argue that, despite its potential role as a coping mechanism against stressful online experience, temporal dissociation induces dysfunctional forms of psychological adaptation that, by inducing addictive behaviours, may also threaten the subject’s task performance on the job [69] and disrupt their capacity to attend to social duties and obligations [70]. 

## 5. Conclusions

Our results contribute to the exiting literature from a theoretical point of view, by investigating the association between stress and SN addiction during the pandemic. They also offer valuable policy insights, highlighting the role of subject-specific variables that can be experimentally manipulated and/or trained to curb temporal dissociation, and thus the risk of addiction. Intriguingly, it was recently reported that emotional regulation ability functions as a mediator between negative affect and internet addiction [71]. Taken together, these findings seem to support the perspective that CBT protocols for internet/SN addiction [72] need to plan treatment interventions focusing on both cognitive (e.g., temporal dissociation) and emotional (e.g., regulation) components of the condition, including physiological stress regulation [73]. Finally, the main limitation of our analysis is that we assessed SN addiction by taking into account several SNs at once. As a direction for future research, further studies are needed to investigate how the interface design and features of specific social networks facilitate or prevent temporal dissociation. Moreover, future studies should explore whether our results also apply to older adults, as technology-related attitudes have been found to be a relevant factor influencing perceived stress and its relationship with smartphone and internet use [74,75,76]. 

## Figures and Tables

**Table 1:** Means (M), standard deviations (SD), and Pearson correlations (r) between variables. Note. *** *p* < 0.001.

	N	M	SD	1	2	3	4
**1. SN Addiction**	312	13.46	4.43	1	0.503 ***	0.292 ***	0.620 ***
**2. Temporal Dissociation**	312	4.91	1.45	0.503 ***	1	0.320 ***	0.843 ***
**3. Perceived Stress**	312	20.6	7.45	0.292 ***	0.320 ***	1	0.248 ***
**4. Cognitive Absorption (Total)**	312	22.47	2.73	0.620 ***	0.843 ***	0.248 ***	1

**Table 2:** *t*-tests results comparing the study variables for males and females (whole sample) on study variables. Mean (M) and standard deviation (SD).

	Male N = 95		Female N = 217				
	M	SD	M	SD	*t*-Test	*p*	Cohen’s d
**Perceived Stress**	17.29	7.10	22.04	7.15	−5.40	0.000	0.66
**Temporal Dissociation**	4.49	1.45	5.09	1.42	−3.35	0.001	0.41
**SN Addiction**	12.78	4.08	13.76	4.55	−1.79	0.07	0.22

**Table 3:** Mediation model predicting SN addiction (N = 312). Coefficient, non-standardized B coefficients; SE, standard errors; CI, bias-corrected and accelerated 95% confidence interval; LL, lower limit; UL, upper limit; PSS, perceived stress score; TD, temporal dissociation; 5000 bootstrap samples. Significant indirect effect in bold. Path coefficient significant at *** *p* < 0.001; ** *p* < 0.01.

		95% CI	
Path Estimates	Coefficient (SE)	LL	UL
a	0.06 (0.011) ***	0.041	0.083
b	1.38 (0.156) ***	1.082	1.695
c	0.08 (0.030) **	0.027	0.146
c’	0.17 (0.032) ***	0.110	0.237
**Indirect Effect**	**Effect (SE)**	**LL**	**UL**
PSS → TD → SN Addiction	**0.086 (0.017)**	**0.055**	**0.123**

## Data Availability

The datasets generated and/or analysed during the current study are available in the Open Science Framework repository: https://osf.io/kjb5n/?view_only=cb03e20ab6bf413aa0cc6ed6277d31a0 (accessed on 20 March 2022).
